# ‘It’s because I like things… it’s a status and he buys me airtime’: exploring the role of transactional sex in young women’s consumption patterns in rural South Africa (secondary findings from HPTN 068)

**DOI:** 10.1186/s12978-018-0539-y

**Published:** 2018-05-29

**Authors:** Meghna Ranganathan, Lori Heise, Catherine MacPhail, Heidi Stöckl, Richard J. Silverwood, Kathleen Kahn, Amanda Selin, F. Xavier Gómez-Olivé, Charlotte Watts, Audrey Pettifor

**Affiliations:** 1 Department for Global Health and Development, Faculty of Public Health and Policy, The London School of Hygiene and Tropical Medicine, London, UK; 2 Wits Reproductive Health and HIV Institute, University of Witwatersrand, Johannesburg, South Africa; 3 MRC/Wits Rural Public Health and Health Transitions Unit (Agincourt), School of Public Health, Faculty of Health Sciences, University of the Witwatersrand, Johannesburg, South Africa; 4 School of Health & Society, University of Wollongong, Wollongong, NSW Australia; 5 Department of Epidemiology, University of North Carolina at Chapel Hill, Chapel Hill, NC USA; 6 Carolina Population Center, University of North Carolina, Chapel Hill, NC USA; 7 Umeå Centre for Global Health Research, Division of Epidemiology and Global Health, Department of Public Health and Clinical Medicine, Umeå University, Umeå, Sweden; 8 International Network for the Demographic Evaluation of Populations and Their Health (INDEPTH) Network, Accra, Ghana; 9 Department of Population, Family and Reproductive Health, Johns Hopkins Bloomberg School of Public Health and JHU School of Nursing, Baltimore, MD USA

**Keywords:** Adolescent girls, Young women, Transactional sex, Sexual exchange, Consumption patterns, Spending patterns, Aspirations

## Abstract

**Background:**

‘Transactional sex’, defined as a non-marital, non-commercial sexual relationship in which money or material goods are exchanged for sex, is associated with young women’s increased vulnerability to HIV infection. Existing research illustrates that the motivations for transactional sex are complex. The fulfilment of psycho-social needs such as the need to belong to a peer group are important factors underlying young women’s desires to obtain certain consumption items and thus engage in transactional sex.

**Methods:**

We use a mixed-methods approach to explore the relationship between transactional sex and consumption patterns among young women in rural Mpumalanga province, South Africa. In the secondary analysis of 693 sexually active young women, we use factor analysis to group the different consumption items and we use multivariable logistic regression to demonstrate the relationship between transactional sex and consumption patterns. The qualitative study uses five focus group discussions and 19 in-depth interviews to explore further young women’s motivations for acquiring different consumption items.

**Results:**

The quantitative results show that young women that engage in transactional sex have higher odds of consuming items for entertainment (e.g., movie tickets) than on practical items (e.g., food and groceries). The qualitative findings also revealed that young women’s perceptions of items that were considered a ‘need’ were strongly influenced by peer pressure and a desire for improved status. Further, there was a perception that emerged from the qualitative data that relationships with sugar daddies offered a way to acquire consumer goods associated with a ‘modern lifestyle’, such as items for personal enhancement and entertainment. However, young women seem aware of the risks associated with such relationships. More importantly, they also develop relationship with partners of similar age, albeit with the continued expectation of material exchange, despite engaging in the relationship for love.

**Conclusion:**

This study shows that young women are willing to take certain risks in order to have a degree of financial independence. Interventions that provide alternative methods of attaining this independence, such as the provision of cash transfers may have potential in preventing them from engaging in transactional relationships. Further, the psycho-social reasons that drive young women’s motivations for consumption items resulting in risky sexual behaviours need to be better understood.

## Plain English summary

Transactional sex is defined as a sexual relationship outside marriage or formal sex work where money or material goods are exchanged for sex. It is associated with adolescent girls and young women’s (hereafter young women) increased susceptibility to HIV infection. Research shows that the motivations for transactional sex are complex. The fulfilment of psycho-social needs such as the need to belong to a peer group are important factors underlying young women’s desires to obtain certain types of goods and to engage in transactional sex. This article advances this line of inquiry by using a mixed- method approach with quantitative and qualitative methods to focus on the relationship between transactional sex and spending patterns of young women in rural Mpumalanga, South Africa and to better understand young women’s needs and wants. The overall findings revealed that young women’s perception of items that were considered a ‘need’ was strongly influenced by peer pressure and that relationships with older men or ‘sugar daddies’ offered a way to acquire goods associated with a “modern lifestyle”, such as items for personal enhancement and entertainment. However, young women seem aware of the risks associated with such relationships and also develop relationships with partners of similar age, primarily for love, but with the continued expectation of material exchange. This study shows that young women are willing to take certain risks in order to be able to have a degree of financial independence. Interventions that provide alternative methods of attaining this independence, such as the provision of cash transfers, may have potential in preventing them from engaging in transactional relationships. Further, the psycho-social reasons that drive young women’s motivations for acquiring items needs to be better understood in order to reduce risky sexual behaviour.

## Background

Transactional sex - defined as a non-marital, non-commercial sexual relationship where men and women exchange sex for material possessions or favours - has been found to be an important contributing factor to HIV vulnerability among adolescent girls and young women (hereafter young women) aged 15–24 in sub-Saharan Africa (SSA) [1–3]. It can place young women at heightened risk of HIV via multiple mechanisms – increasing their number of sexual partners (partner switching to obtain more items) [4–6], encouraging concurrent relationships [4–6] or affecting partner choice [2]. This in turn might bias young women toward older partners who have more resources, but are also more likely to be HIV positive [7–9]. Previous literature has emphasised three paradigms to describe motivations for transactional sex – sex for basic needs, sex for upward mobility and status, and sex for material expressions of love [10]. Furthermore, there are additional psychosocial needs in young women, such as the need to belong to peer groups that motivate them to acquire certain items to enhance their appearance. This may encourage them to engage in relationships in order to obtain these items. In particular, in some instances, young women’s aspirations for a ‘modern lifestyle’, often measured by their consumption patterns (also known as spending patterns) or possession of key consumer goods can drive their pursuit of transactional sex [11, 12]. In settings with high unemployment and where opportunities to acquire these items are limited, studies suggest that by observing older sisters and/or friends, young women realise that material goods can be acquired through sexual exchange [6, 13, 14].

This study advances this line of inquiry by examining young women’s patterns of consumption, their motivations to acquire certain items and the relationship between transactional sex and consumption patterns. Specifically, we explore whether examining young women’s consumption patterns (what they purchased and received as gifts) could yield insights into the dynamics of transactional sex among young women in rural Mpumalanga, South Africa.

### Theoretical underpinnings

To understand the motivations behind young women’s aspirations to acquire specific items, our study draws on theories of belonging and conformity, especially as they apply to adolescents. Erikson [15] describes adolescence as a phase in the life cycle when, “young people are sometimes morbidly, often curiously, preoccupied with what they appear to be, in the eyes of others as compared with what they feel they are...”([16], page 128). Thus, adolescents seek conformity within their groups [16], and are influenced by peers with regard to both neutral behaviours, such as clothing choices, and risky behaviours, such as smoking, drug use and risky sex [17]. The need for belonging forms an important tenet of Maslow’s theory (1943) of hierarchy of needs, which proposes that the need to belong is preceded in importance only by basic biological needs and a desire for safety [18]. Researchers have since found that belonging affects people’s emotions [19], self-esteem [20] and perceptions of others. Importantly, belonging to certain peer groups requires conformity with group values; and a willingness to follow one’s peers [21]. Thus, to be accepted, adolescents may be required to conform to group norms [22]. For example, Wamoyi (2010) shows in her ethnographic fieldwork in Tanzania that young women’s desire for acquiring ‘nice’ things and their readiness to have sex to acquire them, is predominantly shaped by peer expectations and pressure to conform [14]. We follow this line of inquiry in a different context and among a larger sample of individuals. We draw on these theoretical notions to understand young rural South African women’s motivations for engaging in transactional sex.

## Methods

### Research study design

This paper is a secondary analysis of baseline survey data from the HPTN 068 study: a phase III individually randomised conditional cash transfer (CCT) trial in rural South Africa. Data collection was conducted from March 2011–December 2012 in the sub-district of Agincourt in rural Mpumalanga Province, north-east South Africa, an area with high levels of unemployment, poverty and labour migration [23–25]. The intervention involved individually randomising young women aged 13–20 years to receive a monthly cash transfer conditional on school attendance. Study participants were eligible for inclusion in the trial if they were females aged 13 to 20 years; enrolled in grades 8, 9, 10 or 11 at selected schools in the study site; and had a bank or post office account to receive the cash transfer. The total sample size of the main trial was 2533 young women and their parent/guardian (with one young woman per household enrolled). A brief summary of the main trial’s study design is described in Appendix 1; the trial’s sample size calculation, sample recruitment and data collection are described elsewhere [2, 26].

For this study, we used a mixed-methods, sequential explanatory design [27, 28]. Specifically, we explored the relationship between transactional sex and young women’s consumption patterns using quantitative methods. This informed the development of the focus group discussion and in-depth interview topic guides. We then cross-verified findings by comparing the findings of each method, identifying areas where results diverged, converged, or added insights [29].

### Quantitative methods

Of the total sample of 2533 young women, our sample for the secondary analysis included only sexually active, school-going young women (*n* = 693) who reported ever having had vaginal and/or anal sex. For this analysis, the exposure variable was transactional sex and the outcome variable was consumption patterns of young women.

### Measurement tools

Due to the personal nature of some of the questions in the young women’s questionnaire (i.e. details of sexual relationships) young women completed computer-based questionnaires which were primarily self-administered using Audio-Computer Assisted Self-Interviewing (ACASI). Parents/guardians completed interviewer-administered, structured, computer-based household questionnaires. Information on household and socio-economic characteristics (household questionnaire) and socio-demographic background, sexual experiences and partner history (young women’s questionnaire) were included in the tools. Young women filled out questions that were more personal by themselves. Both the parent/guardian and young woman’s interviews were conducted in the language preferred by the participant - in the local language, xiTsonga, or English. The questionnaires were translated into xiTsonga by bilingual researchers and checked for linguistic appropriateness, comprehension and cultural relevance and then back-translated from xiTsonga into English to ensure accuracy and fidelity to meaning.

### Variables

#### Outcome variable

The outcome variable is young women’s consumption patterns, measured by spending patterns on a given set of items. The question in this study asks, *“Over the past month, did you buy for yourself any [item name]* coded as binary (yes/no)” and *“Over the past month, how much money in South African Rands did you spend on [item name] for yourself”.* We used factor analysis to group the 12 items: scented soap, skin creams or lotions; cell phone, airtime, ringtones; shoes, clothing and underwear; cool drinks, chips, etc.; make-up and cosmetics; hairdressing; food/groceries; school uniform or supplies; transport to work and school; beer/alcohol; movies or music tickets and birth control and condoms.

#### Exposure variable

The main exposure variable was ‘ever having had transactional sex’ coded as a binary variable (yes/no) for sex in exchange for money and/or gifts. We asked the young woman about her sexual and relationship history with her three most recent partners, starting with the most recent partner. The four steps carried out to construct the transactional sex variable were:

1. Variable ‘transactional sex for money’ coded 1 if participant said yes to “*Did you feel like you had to have sex with [initials] because they gave you money?”;*
2. Variable ‘transactional sex for gifts’ coded 1 if participant said yes to “*Did you feel like you had to have sex with [initials] because they gave you things (such as airtime, cell phone, groceries, clothes or shoes, perfume or lotions, make-up, cool-drinks, sweets or chips, CDs, DVDs or videos, alcohol or drugs, flowers, other (specify))?”*;
3. Variable ‘transactional sex for money ‘and’ gifts coded 1 if participant had said yes to question (1) “*Did you feel like you had to have sex with [initials] because they gave you money?”* and question (2) “*Did you feel like you had to have sex with [initials] because they gave you things?”*;
4. The final variable ‘transactional sex for money’ ‘and/or’ gifts coded 1 if participant said yes to question (1) “*Did you feel like you had to have sex with [initials] because they gave you money?”* or question (2) “*Did you feel like you had to have sex with [initials] because they gave you* things?”

#### Effect modifiers and other variables

Per capita household consumption (as a measure of household living standards) was hypothesised to be a potential effect modifier in the relationship between transactional sex and consumption patterns. Furthermore, we selected the following variables as confounders in the transactional sex and consumption patterns relationship from our knowledge of the literature and the unadjusted analysis: the age of young women, age of first sex, young women’s employment status, primary caregiver’s educational level, number of household members, orphan status and past year young women’s number of sexual partners. For details on the construction of each variable, please refer to Appendix 2.

### Missing data

Except for the variable *number of sexual partners in the past 12 months* (where missing data were ~ 5%), all the exposure variables had less than 3% missing data. This includes cases where young women ‘refused to answer’. The response ‘don’t know’ was also coded as missing, as the percentage of this response code was small. Only individuals with complete data were included in the final models. Please see Appendix 3 for flowchart on final sample size.

### Analysis

We used exploratory factor analysis to group the 12-item consumption module into sub-groups by examining how underlying common constructs influence individual responses on certain variables [30]. This helped to determine items that “hung together” in a questionnaire; to determine the most important features when classifying a group of items; and to generate factor scores that represent values of the underlying constructs for use in other analyses [31]. Further, descriptive statistics were used to summarise socio-demographic and partnership characteristics of the sample and the prevalence and patterns of transactional sex. Using logistic regression, we estimated the odds ratios for groups of consumption items associated with transactional sex. Unadjusted models were fitted, as well as models adjusted for potential confounders. In this and all subsequent models, we accounted for clustering at the school level by using cluster-robust standard errors.

### Qualitative methods

The qualitative study used a combination of five focus groups (*n* = 25) and 19 in-depth interviews with school-going young women who were participants in the control arm of the main trial. Due to ethical reasons, we could only include young women aged 18–21 years for the qualitative study. Data collection was from November 2012 until March 2013. For the focus groups, young women were selected using the per capita household consumption variable collected in the main survey as a measure of socio-economic status. Based on the information collected, participants were divided into three socioeconomic categories (high, medium and low), for the discussions and allocated to groups of the same category. The per capita household consumption was calculated by summing all household spending and consumption on food and non-food items and by dividing it by the number of household members [32]. A categorical household consumption measure was then obtained by dividing this measure into deciles (1–10). For this analysis, we re-categorised the variable from deciles to three groups for total amount spending/consumption per capita: low (ranging from $1.3 to $15.4), medium (ranging from $15.5 to $32.6) and high (above $32.10).

We used focus groups to explore topics around young women’s relationships and consumption patterns and we conducted participatory exercises to elicit young women’s perceptions of items they consider a “need” versus a “want”. Since young women’s peers were involved in the discussion, focus groups were considered to be effective in stimulating dialogue between respondents and yielding insight into people’s thought processes [33]. To complement the focus groups, we conducted 19 one-on-one private interviews with a subset of young school-going women. Themes that arose in the focus groups, but were sensitive for young women to discuss in a group setting (e.g., sexual relationships) were probed further in the interviews. Ten of the 19 participants invited to be interviewed had reported ever being sexually active and had responded positively to the question on transactional sex. The remaining nine were invited back to participate in the interviews, after the focus group discussion, based on their levels of engagement and responses during the focus groups (we had no prior knowledge of their sexual history). This allowed us to interview young women who were both sexually active and potentially not active.

### Data collection

Focus group discussions were conducted at a village school within the study site over the weekend and were facilitated by a female, xiTsonga speaker and assisted by a designated note-taker. Focus groups were recorded with participants’ permission and later translated and transcribed into English by the group facilitators. The in-depth interviews were 1–1.5 h long, audio-recorded and conducted in a private location. The recordings were transcribed and translated from xiTtsonga to English by the interviewer. Each transcript was quality-checked by a trained third-party researcher to make sure the translation was accurate. Data collection was iterative, with changes made to the topic guide as the focus group discussions and in-depth interviews progressed, based on a review of the data and feedback from the interviewers.

### Analysis

We used thematic content analysis to analyse qualitative data [34, 35]. The first author created an initial coding framework based on the topic guides that was then adjusted deductively and inductively, through frequent discussions between the first two authors. A content matrix was developed to synthesise data and to display emergent cross-cutting themes. All data from these focus groups and interviews were coded using Atlas-ti. Five steps were followed in the analysis of the transcripts: (1) data management and familiarisation; (2) identification of a coding framework; (3) displaying themes and sub-themes; (4) data reduction; and (5) interpretation [27, 35]. Integration of the quantitative and qualitative data occurred in the final discussion; findings that emerged from the preliminary quantitative analysis informed the qualitative analysis. Thematic findings from the qualitative analysis were then compared with the quantitative findings for interpretation.

## Results

### Participant demographics

Table 1 presents descriptive socio-demographic data on the sample (*n* = 693) of sexually active young women. The age range was 13–20 years old (Table 1). Of the whole sample (*n* = 2533), around a quarter reported being sexually active (693 young women or 27.4%) of which 78.2% were 16–20 years old. Among sexually active young women, almost 31% were orphans, with one or both parents deceased and almost 40% reported being worried that their household did not have enough food in the past year. The primary caregiver for most sexually active young women (68.1%) was their mother and a quarter of primary caregivers had never attended school. 78.2% of sexually active young women reported currently having a boyfriend and 21.2% had more than two sexual partners in the past 12 months. 14% (*n* = 97) of sexually active young women reported feeling as though they had to engage in ‘sex for money, gifts or both’ (transactional sex). The majority of transactional sexual relationships were only with the current partner (67%). The majority of items were received from primary partners with 60% receiving money, 25% receiving gifts (such as cosmetics or airtime) and 15% receiving both money and gifts.

**Table 1:** Selected socio-demographic, partnership characteristics and sexual behaviours among sexually active young women (aged 13-20y) (*n* = 693)

Socio-demographic characteristics	Sexually active (column %)
Age of young woman (*n* = 693)	
13–15	151 (21.8)
16–20	542 (78.2)
Per capita household consumption^a^ (*n* = 693)	
Low	220 (31.7)
Medium	279 (40.3)
High	194 (28.0)
Number of household members (*n* = 693)	
2-3members	87 (12.5)
4-5members	233 (33.6)
6-7members	220 (31.7)
> =8members	153 (22.1)
Type of primary caregiver (*n* = 692)	
Mother	471 (68.1)
Father	22 (3.2)
Brother/sister	65 (9.4)
Other blood relative	134 (19.4)
Educational level of primary caregiver (*n* = 692)	
None	176 (25.4)
Primary	196 (28.3)
Secondary	164 (23.7)
Matric or tertiary	128 (18.5)
Adult basic education	28 (4.1)
Orphan status (*n* = 684)	
Parents alive	475 (69.4)
One parent dead	168 (24.6)
Both parents dead	41 (6.0)
Young women’s perceived food insecurity^b^ (*n* = 684)	
No	412 (60.2)
Yes	272 (39.8)
Partnership characteristics and sexual behaviours	
Currently have a boyfriend (*n* = 693)	
No	151 (21.8)
Yes	542 (78.2)
Sexual partners the past 12 months (*n* = 660)	
1	520 (78.8)
2	97 (14.7)
> 3	43 (6.5)
Age of first sex (*n* = 634)	
Before 15 years	127 (20.0)
15 years and above	507 (80.0)
Transactional sex	
Transactional sex (*n* = 693)	
No	596 (86)
Yes	97 (14)
Breakdown of percentages by money or gifts or both^c^ (*n* = 97)	
Sex in exchange for money only	58 (59.8)
Sex in exchange for gifts only	24 (24.7)
Sex in exchange for both money and gifts	15 (15.5)

^a^Measure of household living standards calculated as low ((ranging from $1.3 to $15.4), medium (ranging from $15.5 to $32.6) and high (above $32.10) as monthly expenditures

^b^Young women worried about having enough food for her and her family in the last 12 months

^c^Among sexually active young women who responded yes to question on transactional sex

A subset (*n* = 45) of the sample were invited to participate in the focus group discussions based on their socioeconomic status. Half of these participants were from households dependent on subsistence agriculture and state grants. Half of the participants who were dependant on subsistence agriculture were from female-headed households. Seven of the 19 young women in the in-depth interviews had small children, and two were pregnant at the time of the interview. About third of the 19 young women interviewed worked in informal labour outside school hours, such as in domestic work, yard-cleaning or hairdressing. Twelve of the 19 young women either had fathers who were working elsewhere (temporarily migrated), had passed away, or parents who were separated with the young woman living with her mother. More than half the young women in the overall qualitative sample reported having ever had two or more boyfriends, who were mostly young men within their same age peers. The average female respondent was ~ 2.5 years younger than her most recent male partner.

### Quantitative findings

Exploratory factor analysis produced three groups of consumption items (groups 1, 2 and 3) with overlap between the groups (see Appendix 4). When constructing the consumption variables for analysis, we took into consideration the results of the factor analysis. However, as there was not much variation between groups (i.e. almost all the young women purchased group 1 items and some purchased group 2 and group 3 items), we also took into consideration the sample sizes in each group (which were small for groups 2 and 3) and the qualitative findings when constructing the groupings.

#### Factor analysis groupings

Group 1 (*n* = 630 (91%)) is labelled *‘personal enhancement items’ (PEI)*) as most of the items are for female self-improvement and are considered affordable. It consisted of feminine-enhancement items, such as scented soap, make-up and cosmetics, where expenditures ran up to 200 ZAR (~£18) per month, as well as status enhancing items, such as cell-phone where expenditures extend to up to 600 South African rands (ZAR) (~£50) per month.

Group 2 (*n* = 223 (32.2%)) is labelled ‘*practical items’ (PRAI).* It consists of items that are considered necessary for survival and educational progress, such as groceries, school uniforms and transport to school. These items can be more expensive than Group 1 items. Reported expenditures varied from 10 to 1000 ZAR (~£0.83–83.3) per month for school uniforms and supplies, 10–500 ZAR s (£0.83–41.7) per month for food and groceries and 10–800 ZAR (£0.83–66.67) per month for transport to work or school.

Group 3 (*n* = 216 (31.2%)) is labelled ‘*entertainment and birth control/condoms’* (EBC). It consists of items such as movie tickets, beer/alcohol that are considered items of entertainment. Furthermore, we have included forms of contraception, such as birth control and condoms, that are considered indicators of sexual activity. We grouped these items together using the results of the factor analysis (see Appendix 4: alcohol, movie tickets and birth control/condoms grouped together) and for sample size considerations (the sample number for young women purchasing only birth control/condoms was very small (*n* = 4), hence could not be a separate category). Expenditures varied from 10 to 500 ZAR (£0.83–41.7) per month for movies/music tickets, 10–300 ZAR (£0.83–250 per month) for beer and alcoholic drinks and 10–200 ZAR (£0.83–16.67) for contraception. Some of these items are expensive (such as movie tickets and particular types of expensive condoms that are not provided for free by the government) and some of these items are cheaper (e.g., beer)).

#### Overlap between groups

**Fig. 1:**
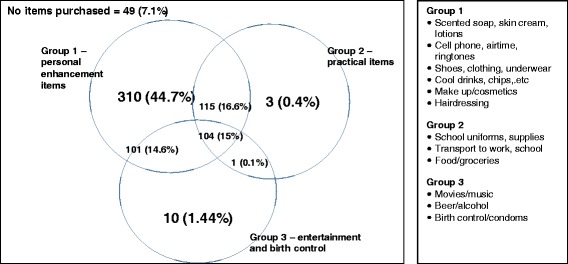
Groupings of items that sexually active young women consume or purchase

Figure 1 shows that young women purchased items from more than one group: a few consumed a combination of groups 1 and 2, and groups 1 and 3; some consumed from all three groups. Most young women consumed group 1 items. If a few young women consumed items from group 2 and 3, they usually had purchased group 1 items too. 310 young women (44.7%) consumed *only* group 1 and only three young women (0.4%) bought group 2 exclusively, as most group 2 consumption is along with group 1 or group 1 and 3. Only ten young women (1.44%) reported consuming group 3 items exclusively. Most group 3 consumption happened along with group 1 or groups 1 and 2.

#### Mutually exclusive groups

**Fig. 2:**
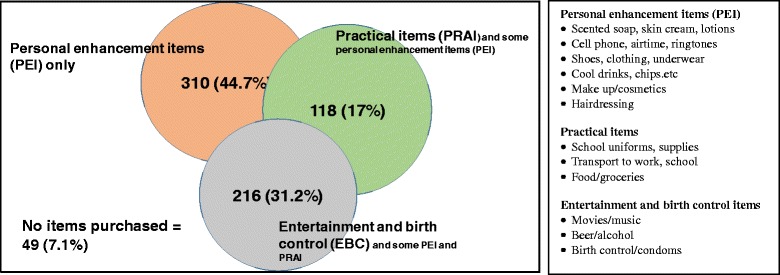
Creation of mutually exclusive groups based on type and value of consumption items among sexually active young women

In order to then create mutually exclusive groups, we used a combination of: results from the factor analysis, our observations from the qualitative findings of young women’s needs and wants (described in the next section), our own theoretical knowledge of the monetary value of items, and our knowledge of the nature of items that implied risky sexual behaviour. The final groups have been illustrated in Fig. 2.

We used the category with the largest sample size (personal enhancement items only) as the reference group for the analysis. Furthermore, as there are a sizeable number of young women who purchased items from groups 1 (PEI) and 2 (PRAI), we have created the category practical items (with some personal enhancement items). Group 3 are young women that are purchasing items for entertainment and birth control/condoms. To create the group entertainment and birth control/condoms (EBC) only that is mutually exclusive, we decided to include young women that are included in group 3 (EBC) only and those that intersect with group 1, with group 2 and with groups 1 and 2. The two consumption variables that were constructed are *‘practical items (and some personal enhancement items)’* and *‘entertainment and birth control/condoms’* items with *‘personal enhancement items only’* as the reference group.

### Transactional sex and consumption of practical items (PRAI) and consumption of entertainment and birth control (EBC) items

In Table 2, we report results from unadjusted and adjusted analyses of the association between young women’s engagement in transactional sex and her consumption of practical items (PRAI) and transactional sex and her consumption of entertainment and birth control/condoms (EBC) items. The results show that engaging in transactional sex is *not* associated with young women’s consumption of PRAI in either unadjusted or adjusted analysis (aOR: 1.09; CI95% = 0.57–2.12; *p* = 0.79).

**Table 2:** Unadjusted and adjusted relationship between transactional sex and young women’s consumption of practical items (PRAI) and entertainment and birth control (EBC) items (compared to personal enhancement items) among sexually active women who reported some consumption^a^

Outcome: PRAI (*n* = 398)^b^	uOR	95% CI	*p*-value	aOR^d^	95% CI	*p*-value^#,^
No	Reference			Reference		
Yes	1.32	0.72–2.44	0.37	1.09	0.57–2.12	0.79
Outcome: EBC (*n* = 486)^c^						
No	Reference			**Reference**		
Yes	1.67	1.03-2.72	0.04*	**3.03**	1.12-8.23	0.03*

^a^As separate analysis for each variable. Sample size for each analysis included in each category

^b^Please note: These numbers are the sum of the number of young women (that are sexually active) who are buying practical items + the number that are buying personal enhancement items. Records with missing data excluded. Total missing: *n* = 30 (7%) These are: work for money (*n* = 8 or 1.2%) missing), orphan status (*n* = 2 or 0.04% missing), type of primary caregiver (*n* = 1 or 0.02% missing), number of sex partners past 12 months (*n* = 19 or 4% missing)

^c^Please note: These numbers are the sum of the number of young women (that are sexually active) who are buying entertainment related and birth control items + the number that are buying personal enhancement items. Records with missing data excluded. Total missing: *n* = 40 (7.5%) These are: work for money (*n* = 7 or 1.3%) missing), orphan (*n* = 9 or 1.7% missing), number of sex partners past 12 months (*n* = 24 or 4.6% missing)

uOR, unadjusted odds ratio aOR Adjusted odds ratio

^d^Adjusted for all confounders – age, number of household members, being an orphan, type of primary caregiver, currently has a boyfriend, number of sexual partners last 12 months, working for money, per capita consumption

^#^*P*-value estimation through likelihood ratio test (LRT); * P-values in bold are statistically significant at the < 0.05 level

Table 2 also shows unadjusted and adjusted associations between young women’s engagement in transactional sex and consumption of EBC items. The unadjusted results suggest that young women who engage in transactional sex have almost 1.7 times higher odds of consuming EBC items than those who do not engage in transactional sex (uOR:1.67; CI95% 1.03–2.72; *p* = 0.04). We hypothesised that household per capita consumption (a measure of living standards) might be an effect modifier for the relationship between transactional sex and consumption of EBC items. To test this, we conducted a test of interaction. The results showed that overall there was almost no interaction (*p* = 0.18) between household per capita consumption and transactional sex and consumption of EBC. Furthermore, after adjusting for confounders, there was evidence to show that young women who engage in transactional sex have three times higher odds of consuming items for EBC items (aOR:3.03; CI95% 1.12–8.23; *P* = 0.03).

### Qualitative findings

#### The perception of “needs” and “wants”

To study the motivations for transactional sex, we sought to understand how the perception of ‘needs and wants’ related to consumption patterns of young women. ‘Needs’ were defined as items that a person cannot survive without; ‘wants’ were items that a person desires, but can live without. During the focus groups, young women were asked to freely list on a whiteboard, items that themselves and their peers were either spending money on or what they sought to acquire. They were then individually asked to categorise items by affixing a red (need) or blue (want) sticker next to the items on the whiteboard. Items were then grouped into four categories based on their types, value and utility: personal enhancement items, practical items, expensive items and entertainment items. Table 3 lists the results.

**Table 3:** Needs and Wants categories

Groupings	Items	Need	Want
Personal Enhancement Items	Expensive perfume	Need	
	Hair extensions	Need	
	Lingerie (fancy underwear (such as *Jockey*)	Need	
	Toiletries (scented soap, skin cream, body lotion, powder, roll-on deodorant)	Need	
	Make-up (eyebrow pencil, mascara, false eye lashes, eye liner)		Want
	Cosmetics (Moisturising cream, nail polish (Cutex), skin-brightening cream, lip balm, bag, hair control)		Want
	Salon treatment (hairdressing/hair highlighting, relaxing, dyeing, manicure, waxing)		Want
	Accessories (watches/handbag)		Want
	Jewellery (bracelets, earrings, necklace, gold tooth)		Want
	Body piercings (belly-ring nose-ring, tongue-ring, tattoo)		Want
Practical Items	Clothes	Need	
	Ordinary cell phone and airtime	Need	
	Shoes	Need	
	Female items (underwear, sanitary pads)	Need	
	Food, groceries	Need	
	School uniforms/supplies	Need	
	Transport to school/work	Need	
	Birth control/condoms	Need	
Expensive Items	Expensive clothes (skinny jeans, *hlokoloza* (mini skirt), *tekkies* (trainers), branded clothes	Need	
	Fancy shoes (high heels, C*arvela* (expensive Italian branded shoes)	Need	
	Expensive phones (blackberry/camera phone/Nokia/×2)		Want
	Expensive foods (chocolate, yoghurt, grapes, restaurant)		Want
Entertainment/leisure	Beer/alcohol		Want
	Cold drinks and chips, *Ultramel* (ready-made custard)*, Lays* (potato crisps)		Want
	Movies/music and travelling		Want
	Pocket money		Want

The results in Table 3 show that ‘needs’ included practical items (e.g., clothes, shoes, sanitary pads), female personal enhancement items (e.g., lingerie, hair extensions) and certain expensive items (e.g., fancy shoes, expensive clothes). When probed on whether phones or perfume were actual needs, responses indicated that they were considered unaffordable needs; reasons ranged from needing to conform *(‘because nowadays other people are using Blackberry’)*, to needing to boosting their status *(‘perfume is attractive to me because when I smell it from someone smelling good, like from teachers, then I tell myself that it’s an expensive perfume, so that is why it becomes attractive to me’)*. Some young women showed efforts to practice safe sexual behaviours by mentioning birth control and condoms as needs *(‘We want to protect ourselves from illness, because you cannot grow up without doing it, so you need to protect yourself’*).

‘Wants’ included some personal enhancement items (e.g., cosmetics, jewellery), entertainment/ leisure (e.g., beer/alcohol, movie tickets), as well as some expensive items, (e.g., expensive phones). Reasons given for wanting these items were to be able to identify with certain role models either within their peer group as illustrated by this quote:

*P3: Even if you can bath and wear nice clothes but if you didn’t put make-up aah…you are not really (young woman’s name].*

*P4: It is just because most of the young women nowadays they like fashion, that is why if you don’t have make-up you will not feel good.*

*Focus group 4, high socio-economic status*

Some items were classified both as a ‘need’ and a ‘want’ based on their perceived value. For example, staple food (e.g., maize) was a need and expensive food items (e.g., chocolate) were considered wants. Interestingly, “ordinary clothes and shoes” as well as “expensive clothes and shoes” were both considered needs. Furthermore, owning expensive clothes were represented by many participants as ‘needs’ similar to the need for food. Hence, it is the ‘perception of need’ rather than an ‘actual need’ that is important, as from the perspective of these rural young women, owning expensive clothes or expensive shoes is construed as a necessity in their lives. It therefore appears that young women are motivated by materialistic desires, as well as the pressure to conform to peer group expectations through adopting symbols of sophistication and a modern lifestyle.

However, some young women acknowledged the differences between expensive and basic items. They recognised that coveting unaffordable items did not imply need and that the perception of needs (and wants) is relative and based on an individual’s household socio-economic status.

*I: I heard someone say that Blackberry is unnecessary. Can you please tell me why?*

*P2: Blackberry is too expensive and I’m still a school child, and parents cannot afford to buy me Blackberry. I will be fine if I can have cell phone worth R150 [£9]. The most important thing is to communicate.*

*P3: It is necessary to have Blackberry, because you will chat for free on Facebook and Blackberry messenger.*

*P1: It is not necessary because even if you don’t chat it doesn’t mean that you are not a human being.*

*P3: That is why I said my need is not your need.*

*Focus group 4, high socio-economic status*

### The role of peer pressure and socio-economic status

Peer group pressure played an important factor in influencing young women’s perceptions that certain items were ‘needs’ and motivating them to acquire such items. This pressure had many components. The first was to identify with certain role models within their peer group:

*P3: Even if you can bath and wear nice clothes but if you didn’t put make-up aah…you are not really [role model’s name].*

*P4: It is just because most of the young women nowadays they like fashion, that is why if you don’t have make-up you will not feel good.*

*Focus group 4, high socio-economic status*

But possessing certain expensive items enables a young woman to send a signal to her peer group that she is a ‘cool, modern woman’, making her the object of envy among her peers, and in turn enhancing her self-esteem.

*P: [I want these items] because they are needed and that everyone wish they can have it...that if I found myself having this I will be ‘the’ person among the people and when people see me coming from there they will turn their heads and look at me.*

*In-depth interview 2, aged 21y*

However, it appears that many of the perceived needs are also about social standing among peers. For example, wearing nice uniforms are necessary to avoid being teased and humiliated by classmates, more than being ‘real’ barriers to school attendance. This did not seem to differ between different socio-economic focus groups, as illustrated by the two quotes below:

*I: Ok. What happens if you do not have one of the items that you need?*

*P2: Like school uniform, I won’t go to school without enough uniform. And when I wear a skirt that is tearing [torn] and then my friends laugh at me.*

*Focus group 3, low socio-economic status.*

*P1: Like if you go to school you need to wear proper school uniform because the school children will laugh at you.*

*P3: Even if you can bathe and wear nice clothes but if you didn’t put make-up aah…you are not really (certain role model in the village).*

*Focus group 4, high socio-economic status*

Status among friends and perceived “ranking” within a social order was of importance to young women.

*P1: We want to make ourselves beautiful so that people can recognise us.*

*P3: That’s what I was saying if you didn’t put make-up people say you’re a traditional girl [I:we laughed], if you have put make-up people recognise you that you are South African.*

*P1: Because people from Mozambique they don’t put make-up, so if you don’t put make-up they will compare you with them.*

*Focus group 4, high socio-economic status*

Overall, in the in-depth interviews there was not much variation by socio-economic status in the responses around needs and wants, except for in a couple of interviews where the young women recognised the constraints of their household’s socio-economic situation. They acknowledged that despite having desires, the items were unaffordable, but they were hopeful for the future. These have been illustrated by the following excerpts:

*P: If you use make up it wastes your time, some years back at school l could see that something was wrong about me using make up. You find that we are in class at school and people have put their make up; you have finished your make-up and you don’t have money to buy. You will feel like you are very poor. So l stopped using make up because it was not good to use make up when you are still a student.*

*I: How do they (make-up) make you feel?*

*P: It made me feel like other children because I can see that I’m different to them, but I feel ok about the way l am. l believes that one day l will be successful.*

*In-depth interview 14, aged 19 years*

*P: I don’t care even if I don’t have Cutex (nail polish) ...but my heart is painful when I go to school and didn’t braid my hair and others will laugh at me saying that I’ve aged......Even if they don’t laugh at me they gossip about my hair.....I have an afro hair so I just braid it without using hair extension.. I feel good (when they gossip) because I told myself that whom are they going to gossip about, if they don’t gossip about me. You must accept because people are gossiping.*

*In-depth interview 14, aged 19 years*

### Funding consumption patterns

#### Parents

For many young women (*n* = 18), particularly in the focus groups, parents or primary caregivers paid for items considered needs or ‘practical items’, such as transportation to school, as well as items of female personal enhancement and hygiene that require regular monthly replenishment, such as roll-on deodorants. Some young women also indicated that parents, on occasion, bought their daughters hair pieces and earrings. Items that parents did not pay for were mostly entertainment-related, such as movie tickets, beer/alcohol or expensive phones or expensive creams.

In particular, young women felt that their parents were traditionalists, compared to their own ideas of modern living, as shown by the following quote on the modern-day use of body creams compared to traditional items that had been used by parents:

*P3: And our parents say that when they were young they were not using fancy cream that smells good they were using “Nhlampfura” (Oil of nut or fruit) in older days… [I: they laughed] and why do they have to spend money for us on fancy creams whereas in older days they were using “Nhlampfura” and men proposed them even if they don’t smell good….*

*Focus group 2, high socio-economic status*

When probed in the focus groups on how young women from higher socio-economic households obtain items, the general response irrespective of the socio-economic status of the group was that parents from these households have the means to afford non-essential items when compared to those from poorer households. Some participants mentioned the choices that young women from richer households have in terms of the security of family support and the ability to demand something and have these desires met. This, as a result, makes them more confident (when compared to young women from poorer households).

*P2: And some our situation is different it might happen that my friend is coming from the richer family and I from poorer family and they will able to buy her fancy things and at home they will not afford to buy me something because they cannot afford it because we don’t get things easily because of not having money.*

*Focus group discussion 2, high socio-economic status*

In contrast, parents from poorer households only bought items they considered essential, such as food and clothes. Thus, the budget constraint in low socio-economic households dictated items considered essential by parents.

*P: Like when their mom got a piece [part-time] job from other households, like to sweep the yard then when she get paid she buy these items like food for their children and things that they can make them look able [healthy].*

*Focus group discussion 3, low socio-economic status*

However, not all poorer families bought their children only essential items. For example, a young woman from a low-income family described how, in her friend’s family, the use of cosmetics was not encouraged, as her parents perceived it to be something which would attract untoward attention. However, they did encourage cultivation of an aesthetic sense and provided the young woman with nicer clothes to help her fit in. Young women noted that parents were aware of their need to maintain status. But, many of these families were supported by social grants and did not have the financial means to buy ‘items of need’. This resulted in young women resorting to other ways of obtaining items, as illustrated by the following quote:

*P5: Because like when they buy them make-up they will think she will start to be a prostitute.*

*I: Mmm…Ok, so what about those who like it?*

*P3: They want their children to be beautiful and be recognised.*

*Focus group discussion 3, low socio-economic status*

#### Sugar daddies

‘Sugar daddies’ were mentioned as sources of funding for items parents did not provide. When probed on the definition of a sugar daddy, the response was: “an older person (>10 years older), more established in his career, more financially stable that solicits younger women primarily for sex*.*” During focus groups, the discussion related to sugar daddies was not positive; there was open acknowledgement of the associated social stigma associated with sugar daddy relationships and the fact that that sexual relationships with sugar daddies place young women at risk of contracting diseases. Young women acknowledged that (other) young women chase sugar daddies and stated that hardships at home compel these young women to engage in such sexual relationships. The hope was that sugar daddies would help them afford items that would enhance their appearance.

*P2: Because youth want expensive thing and parents won’t afford it like hair-piece, a parent won’t take you to the salon to do your hair, but if you are involved with an old man he can be able to take you to salon and do your hair.*

*Focus group discussion 2, high socio-economic status.*

*P2: Sugar daddies have money and I want their money… [I: we laughed]. So nowadays girls want to make themselves beautiful, look nice and wear nice clothes. Some other girls when you give them money they go to the shop and buy snacks… [I: we laughed] but look at me I eat well because I have a relationship with a sugar daddy.*

*Focus group discussion 5, low socio-economic status.*

In contrast, during in-depth interviews, young women were more reticent in their responses when probed about their personal engagement with sugar daddies. This might be because young women in the interviews were asked questions related to their personal situations, compared to the focus groups, when the discussion was more general. There was agreement that the desire to be with a sugar daddy arises from materialistic motivations and peer pressure.

*P: [I don’t want sugar daddies] because he is too old and on the other hand I abuse my body. ….. Some they want clothes and expensive cell phone like ‘Samsung Galaxy’ [expensive smartphone]. You find that they like it and sometimes this is cause by peer pressure. You find that my friends are having a relationship with a sugar daddy and she had sex with him then he buys her some items or expensive clothes like label clothes or an expensive cell phone. I like it then I start to involve myself [with a sugar daddy] to get the items that I need.*

*In-depth interview 10, aged 20y.*

Yet, there was recognition, even in the focus groups that any sexual relationship with a sugar daddy placed young women at risk of abusive behaviour and of contracting HIV, as detailed here:

*P: Sugar daddy is a person who has a family. If you are in relationship with him is like he is abusing you because he is older than you and has his wife.*

*In-depth interview 11, aged 19y.*

*P1: Like you find that she’s in a relationship with a sugar daddy and you don’t know his [HIV] status and he’s just buying her so he can sleep with her and leave her with diseases.*

*Focus group discussion 3, low socio-economic status*

#### Boyfriends

In contrast to sugar daddies, there was a perception that *“a boyfriend is a person you love. A sugar daddy is a boyfriend with benefits...like airtime. In-depth interview 6”, age 21).* The perception was that receiving money or gifts from a sugar daddy can almost be likened to prostitution, as the expectation of sex with the provision of money or gifts is overt, whereas receiving gifts or money from boyfriends of similar age did not have the same level of explicit expectation of sexual exchange. Boyfriends’ provision of financial and material support was mentioned extensively by young women in both focus groups and interviews as a reason for becoming involved in relationships. Some focus group participants openly expressed their preference for being with a partner of similar age, but commented that the likelihood of these men having a lucrative job was slim, hence were less able to act as providers. Similarly, as most young women were young and inexperienced, they aspired for a ‘perceived better life’ filled with potential, such as with a man who can provide. Thus, aspirations to “fit into the crowd” drove their desire to engage in relationships. Some young women mentioned that receiving gifts help increase their social acceptance among friends. They did not report feeling obliged to have sex with their boyfriends in exchange for these items *(“he buys me things that I should have it as a girl”, In-depth interview 8,aged 20y).*

*P: [the reasons I am with him] are because I like things… it’s a status. He buys me airtime. I call him and friends. And boast on them that I have been bought airtime. I feel good, something like that.*

*I: What would you do if he didn’t buy you things you want like airtime?*

*P: I will just leave him, why doesn’t he buy me airtime it will mean he buy it for someone else…*

*In-depth interview 17, aged 20y.*

*P2: Yes it happens. To have a relationship with someone who own a nice car because when I am sitting with my friends I will tell them that I ride with a nice car. And then I will influence them not to have relationships with school boys. They have to date those who are working.*

*Focus group discussion 1, low socio-economic status.*

In the focus groups, when probed on motivations for young women to be in relationships, two-thirds of young women explicitly said that it was for money or material goods. The remainder of young women in the focus groups were more reticent about money being their main motivation. Privately, in the interviews, most young women were likely to back away from group consensus to say that it was love that made them engage in sexual relationships with men. Nevertheless, even though love featured in most young women’s responses, there was a clear, but implicit understanding that the need for money and gifts were important in all relationships. It appears from this data that the rules and norms surrounding sex in exchange for money/gifts are intricate and sometimes ambiguous; gifts and money are important even in relationships characterised by love. Yet, money exchange does have one constant: a sexual relationship does not exist without the expectation of a male-to-female transfer of money or gifts. But, young women did make the distinction between “being opportunistic” and “feeling provided for and looked after”.

*I: Mmm… the one you had a relationship with, where did you meet him?*

*P: At school. I wanted him to help me. Like when I need money to use he was able to help me things like hair extensions. When I wanted it he was able to give me money to buy it… I was feeling happy about it [receiving money].*

*I: Tell me did you feel like you had to have sex with him to receive money or gifts.*

*P: No. It was the issue of love.*

*In-depth interview 18, aged 20y.*

Overall, it is important to mention that social pressure demands that young women speak of relationships as being ‘for love’, and while this is certainly a key element of their relationships, the potential for material gain is also a significant component. Few young women felt that they had to have sex because of receiving gifts, but gift giving was still considered an essential component of their relationships.

## Discussion

This is one of the first and only studies to quantitatively explore the relationship between transactional sex and consumption patterns in a cohort of young women in rural South Africa. Our quantitative results showed that transactional sex was associated with almost a three-fold increased odds of young women consuming items for entertainment (e.g., movie tickets), items that are considered indicators of risky sexual behaviours (e.g. alcohol) and items that are indicators of sexual activity (e.g., birth control and condoms), but this did not vary by household socio-economic status. There was, however no evidence of an association between transactional sex and young women’s consumption of practical items, such as food, school uniforms and transportation to school. Furthermore, our qualitative data offers unique insights into young women’s classification of items that are considered ‘needs’ versus ‘wants’. It is one of the first studies that links these qualitative conceptualisations to young women’s consumption patterns using the framework of Maslow’s hierarchy of needs to categorise and understand young women’s needs and wants. It suggests that the distinction between ‘need’ and ‘want’ is ambiguous and that motivations for obtaining such items are not driven merely by survival or consumerism, but by higher order psycho-social needs such as the need to belong to a peer group. These results illustrate that Maslow’s hierarchy of needs does not apply strictly to young women’s needs and wants. In situations of considerable poverty, unstable household structure, scarce economic opportunities and a rapidly globalising economy, young women in rural South Africa have ‘admitted’ needs (for items that are considered necessary, such as food or clothes) and ‘hidden’ needs (such as for expensive clothes, cosmetics) that are their ‘wants’. Hidden needs appear to play a pivotal role in these young women’s quest for enhancing their self-esteem and status and feeling like they belong to and are accepted by their peer group. This aligns with Leclerc-Madlala’s (2004) research with unmarried young women aged 15–25 in an urban township of Durban, South Africa. Madlala’s findings showed that the practice of transactional sex is best illustrated by a continuum, where rewards or gifts can vary between what are generally perceived to be ‘needs’ and what are generally understood as ‘wants’. However both are always represented and expressed as ‘needs’ [36]. Further, our study illustrated that the pursuit of expensive or personal enhancement items was not just a mere exercise of consumption, but one that is perceived to be necessary for *survival* from social exclusion or loneliness. As articulated by Stoebenau et al. (2013) the term “economic vulnerability” initially coined by Kuate-Defo captures relationships that are motivated by “survival” as well as conformity, social status and pride among peer group members [37]. Thus, in contrast to Maslow’s framework, where a sense of belonging is linked to the development of self-worth and recognition, one can infer that young women’s notions of survival extend beyond just biological and physiological needs (food, clothing, shelter) and that the need for a sense of belonging to their peer group, and for self-esteem are perceived to be essential to their survival. Hence, the findings suggest that transactional sex linked to subsistence or to consumerism are not necessarily mutually exclusive. This aligns with research in South Africa by Adato et al. (2016) and in Kenya by Mojola (2014) that demonstrates how issues related to the articulation of gendered needs (e.g., deodorants, skin creams) and non-material needs (e.g., peer pressure, social status and conformity) shape young women’s decisions to engage in transactional sex [38, 39].

This study also showed that young women aspire for a variety of items, but their household economic situation, along with circumscribed economic opportunities in the area, imposes spending constraints. In turn, relationships appeared to fill this funding gap for young women’s consumption. This was especially the case when parents were not willing or able to provide an extra allowance to obtain items that are considered ‘non-essential’. This aligned with Allison Pugh’s (2009) research in the United States demonstrating the commodification of childhood in the United States and the dilemma facing low income parents when dealing with their children’s growing materialistic desires [40]. According to Pugh, these parents had clear spending constraints, hence only bought essential items and maybe indulged their children occasionally, despite being empathetic to their children’s needs and wants [40]. As a result, some young women enter similar age relationships with boyfriends or become involved with sugar daddies to have a steady conduit through which money or gifts flow. Participants (particularly in the focus groups) frequently mentioned the existence of sugar daddies and that (other) young women have relationships with them either to fulfil basic needs, but more often to satisfy their consumerist desires. Interestingly, none of the participants admitted to personally engaging with sugar daddies when probed in the in-depth interviews. There was a negative connotation associated with sugar daddies as a person who was ‘abusive’, was substantially older and disrespectful towards young women. There was recognition that in a context of economic deprivation, sugar daddies were a source by which young women could access things they might not be able to afford otherwise. In addition, in a setting where awareness of diseases, including HIV infection, is high; many young women mentioned that sugar daddies would put them at risk of disease, along with the social stigma associated with engaging with one. As has been shown in the literature, young women engaging in sexual relationships with older men are considered to be at much higher risk of HIV acquisition compared to a young woman engaging with a similar age male peer [41, 42]. Thus, overtly engaging in sexual relations for money or gifts with a sugar daddy was considered closer to sex work, as the negotiation in the exchange was explicit, whereas having a relationship with a similar age boyfriend (even if it is a source for money or gifts) was still nuanced in its meaning and significance. This finding indicates that relationships with sugar daddies are not necessarily rampant as previously suggested [43] and that young women were aware of the associated risks. Further, as shown by other research conducted by this study authors using the same sample of young women [44], similar-age relationships appear to be more the norm, even if there was a transactional component.

Related to this, our qualitative results show a far more nuanced relationship between sex, love and the provision of gifts. Women want to be the object of love (and desire) from their similar age male partners, but also expect to receive money or gifts as an expression of this desire. This has also been discussed in multiple studies throughout the sub-Saharan African region (South Africa, Malawi, Tanzania) [45–48] that demonstrate the degree to which love and money are tightly intertwined in relationships. A number of young women rationalise their behaviour of coveting material items or gifts and genuinely believe that they are in the relationship for love (that they engage in sex for love). If they have a boyfriend, they do expect either money or gifts, but in all these cases the implication is love and not sex for gifts/money [44].

From our quantitative results, as transactional sex is associated with the consumption of entertainment related items, such as alcohol, an argument could be made based on the literature that young women who engage in the “game” of alcohol-sex exchange may to some extent use it as a form of entertainment, both in the procurement of alcohol and related socialisation that occurs in these venues [49]. Research by Watt et al. in South Africa suggests that alcohol-serving venues provide a space to foster social identity with peer groups and to deal with boredom that comes with circumscribed employment opportunities and lack of recreational activities [50, 51]. Other research in similar settings has shown that gender inequality and poverty provide a context in which the alcohol-sex exchange can become an attractive strategy for young women [52].

A theme that also emerged from the qualitative research is that young women seek boyfriends out of an aspiration for social mobility, economic independence or simply a life enhanced by status, including expensive clothes and fancier items. Our quantitative results complemented this picture by showing that engagement in transactional sex does not vary by socio-economic status. In fact, rural young South African women are engaging in transactional sex to also purchase entertainment items, which suggests that young women are aspiring for a lifestyle, which is not just about dire need, but also about choice and entertainment. This finding also aligns with research from other settings in South Africa, Zimbabwe and Kenya [53–56] that also discuss the commodification of young women’s consumption patterns and the role of transactional sex. Further, in the context of post-apartheid South Africa, through the lifting of restrictions of Black people’s movements, improved road infrastructure to rural areas, the influx of goods from other counties and increased access to visual and print media, previously “remote” rural areas in South Africa have gradually opened socially and geographically. These macro-level changes have played a role in exposing young women to globalised images and availability of goods [11]. In addition, one could also argue that there are different role models for this generation than there might have been for previous generations. During apartheid, there was not the same access to images of successful Black people as there are now, which makes it easier for young Black women to identify with role models [11]. Thus, irrespective of young women’s household socio-economic position, aspirations for items might also be influenced by access to media and advertisements, peer groups, globalisation or macroeconomic changes in the country, television shows and a strong desire to be socially accepted.

### Strengths and limitations

This study has a number of strengths and limitations. The use of laptops and ACASI software for data collection allowed for increased privacy in the quantitative survey. By removing the face-to-face component of surveys, the social-desirability aspect of responses was partially addressed, thereby tackling the potential issue of over or under-reporting of sensitive questions, such as transactional sex. To address the social stigma around the reporting of sexual behaviours, one-on-one interviews were conducted in a private setting, such as the participant’s house, and participants were assured that the research was going to benefit the community and that interview data would be kept anonymous. We found that focus group discussions and in-depth interviews complemented each other well, as the focus groups permitted free flow of information in a group setting. Young women were less hesitant to speak about the sexual behaviours, as they were explicitly asked to talk generally in the focus groups. In fact, they were more open discussing sugar daddies in the focus groups than in the in-depth interviews. Thus, the larger themes around their motivations to have a sexual relationship were better explored privately in the interviews.

This analysis is however limited by the sample for the qualitative data, which limits our ability to extrapolate these results to other individuals, including young women that are under 18-year olds and/or do not attend school. Moreover, eligibility criteria for being part of the main trial (that young women and their parents/guardians had to have documentation to open a bank account), meant that poorer young women who have limited access to basic services and social grants could not enrol in the study. It may be that transactional sex in this subset of disadvantaged young women is less consumerist-oriented and more subsistence-driven. Hence the results need to be interpreted with caution. Further, we recognise that birth control and condoms are separate items and have different meanings in terms of their use; birth control methods do not require negotiation between partners and the choice of their use is with the young woman, whereas, condoms require up-front negotiation between partners, resulting in a potential loss of control for young women. However, as this was a secondary analysis of survey data, and both the items were already together, we are not able to separate them out. Hence, for this analysis, we have considered them together as birth control/condoms. It is also important to mention that in South Africa, condoms are distributed for free by the government Department of Health and dispensed by local authority clinics. [57] Thus, young women might not need to engage in transactional sex in order to obtain condoms. However, there was a perception among young people that government provided free condoms were of lower status and quality [58] resulting in young women preferring the fancier, more expensive condoms. The government has recently embarked on a new campaign to provide free condoms in school in order to increase availability, but also re-brand them, so that they appeal to young people. This should hopefully increase the uptake of free condoms [58].

Finally, due to time and monetary constraints, we were unable to interview boys or men and get their perspectives on young women’s motivations for being in relationships. Hunter’s (2010) historical analysis of love and masculinities in rural KwaZulu-Natal [45] and Bhana and Pattman’s research [46] in a township outside Durban, KwaZulu-Natal discusses provider roles as an important feature in feelings of masculinity in South African men and how it frames their relationships with women. Future research in the same study site should explore men’s perspectives to compare responses and enrich these findings [45, 46]. Moreover, the cross-sectional nature of the data means that we cannot make any claims about the causal direction of these relationships, preventing us from understanding to what extent consumption *motivates* sexual behaviour. For example, young women reporting transactional sex are using money for entertainment-related items; entertainment might be a consequence of the transactional relationship rather than a reason for it. We have tried to address this limitation through qualitative work where questions during in-depth interviews explore the types of items young women consider a need and want and the motivations for obtaining such items. Recall bias is also a potential issue, as the variables to measure transactional sex, young women’s consumption patterns and all the potential confounders are self-reported. Question time-frames were chosen to be consistent with other studies (where applicable) and to facilitate recall (e.g., sexual partners over the past 12 months or past month consumption).

## Conclusion

This study offers evidence of an association between transactional sex and consumption patterns among young women in rural South Africa. It demonstrates that young women who engage in transactional sex have higher odds of consuming items for entertainment that might also lead to risky sexual behaviours. Thus, an underlying driver for transactional sex is the need to close a gap in the funding of certain consumer-oriented items. However, the qualitative findings also suggest that young women seem aware of the risks associated with sugar daddy relationships. Relationships between young women and men of similar age appear to be more prevalent than sugar daddy relationships. In these relationships, the male to female exchange of commodities is common and expected, but not explicitly linked to sexual activity. The motivations given for the acquisition of specific lifestyle-related items suggests that they were not related either to subsistence or consumerist desires, but to the fulfilment of psycho-social needs for belonging, peer acceptance and self-esteem. This study shows that young women are willing to take certain risks in order to be able to have a degree of financial independence. Interventions that provide alternative methods of attaining this independence, such as the provision of cash transfers, may have potential in preventing them from engaging in transactional relationships [59]. However, the psycho-social reasons that drive young women’s motivations for consumption needs within a rural, but globalising context needs to be better understood. In particular, peer-led education programmes have shown to be capable of providing vulnerable youth with psychosocial support, as well as information and decision making skills [60]. These programmes will also leverage youth resilience and protective skills within the confines of difficult economic and social circumstances to allow them to successfully navigate safer sexual relationships [61, 62].

## Figures and Tables

**Fig. 3:**
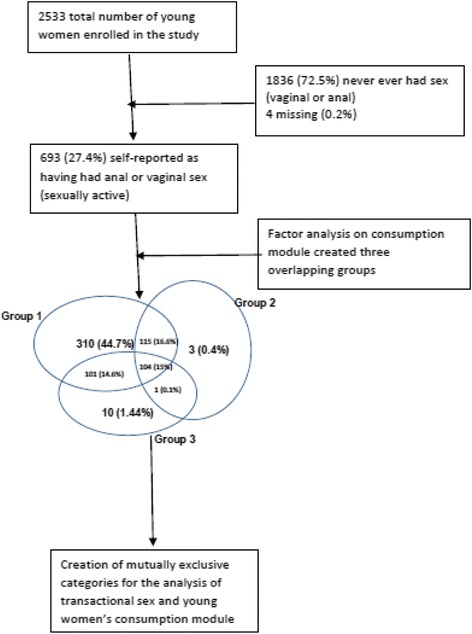
Flowchart for analysis between transactional sex and consumption patterns

**Fig. 4:**
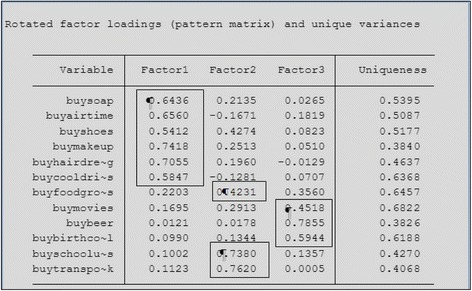
Factor loadings for 12 items from consumption module

## Appendix 1

### HPTN 068 trial baseline sztudy design:

Data collection was conducted from March 2011–December 2012 in the sub-district of Agincourt in rural Mpumalanga Province, north-east South Africa, an area with high levels of poverty, unemployment and labour migration [23–25]. The Medical Research Council (MRC)/Wits University Rural Public Health and Health Transitions Research Unit runs the Agincourt Health and Socio-Demographic Surveillance System (AHDSS) in this area and this was the platform for identifying eligible households and young women [52]. The trial sought to explore whether providing cash transfers to young women and their households - conditional on school attendance - reduces HIV incidence among young women [26]. The intervention involved individually randomising young women aged 13 to 20 to receive a monthly cash transfer, conditional on school attendance. Control participants received no cash transfer but attended annual study visits. Study participants were eligible for inclusion in the trial if they were females aged 13 to 20 years; enrolled in grades 8, 9, 10 or 11 at selected schools in the AHDSS study site; and had a bank or post office account to receive the transfer. The participants were excluded if they were pregnant or married at baseline. Both parental/legal guardian consent and young woman consent/assent were required to participate.

## Appendix 2

### Construction of effect modifier and confounding variables

- *The age of young women*, was recorded as a continuous variable from 13 to 20 years and was re-categorised into two groups of 13–15; 16–20 for equal sample size in each category.
- *The age of first vaginal and/or anal sex* was constructed from the questions *“how old were you when you first had vaginal sex? How old were you when you first had anal sex?”* and re-categorised into two groups - < 15 years and 15 years and older.
- *Employment status of the young woman* was recorded as a binary variable and constructed it from the question *“Did you do any work for pay or family gain, including payment in kind such as food or housing?”*
- *Educational level of primary caregiver* was measured as a categorical variable with four categories: none, primary, secondary, matric (year 12) and adult basic education.
- *Orphan status* (defined as either one or both parents deceased), was binary and constructed from the question, if the mother was alive and if the father was alive.
- *Young women’s number of sexual partners in the past 12 months* was recorded from 0 to 15 and was categorised into 4 groups: 0, 1, 2, > 3.
- Per capita *household consumption* as a measure of living standards was calculated using the module on food and non-food spending and consumption in the household questionnaire. This was done by summing all household spending and consumption on food and non-food items and by dividing it by the number of household members (total spending and consumption per capita) [32]. A categorical household consumption measure was then obtained by dividing this measure into deciles (1–10). For this analysis, we re-categorised the variable from deciles to three groups for total amount spending/consumption per capita: low (ranging from $1.3 to 15.4), medium (ranging from $15.5 to 32.6) and high (above $32.10).
